# 3D shape reconstruction of the femur from planar X-ray images using statistical shape and appearance models

**DOI:** 10.1186/s12938-023-01093-z

**Published:** 2023-03-24

**Authors:** Daniel Nolte, Shuqiao Xie, Anthony M. J. Bull

**Affiliations:** Department of Bioengineering, Imperial College London, London, SW7 2AZ UK

**Keywords:** 2D–3D reconstruction, Orthopaedic reconstruction, Digitally reconstructed radiographs, Anatomical measures, Bone loss

## Abstract

Major trauma is a condition that can result in severe bone damage. Customised orthopaedic reconstruction allows for limb salvage surgery and helps to restore joint alignment. For the best possible outcome three dimensional (3D) medical imaging is necessary, but its availability and access, especially in developing countries, can be challenging. In this study, 3D bone shapes of the femur reconstructed from planar radiographs representing bone defects were evaluated for use in orthopaedic surgery. Statistical shape and appearance models generated from 40 cadaveric X-ray computed tomography (CT) images were used to reconstruct 3D bone shapes. The reconstruction simulated bone defects of between 0% and 50% of the whole bone, and the prediction accuracy using anterior–posterior (AP) and anterior–posterior/medial–lateral (AP/ML) X-rays were compared. As error metrics for the comparison, measures evaluating the distance between contour lines of the projections as well as a measure comparing similarities in image intensities were used. The results were evaluated using the root-mean-square distance for surface error as well as differences in commonly used anatomical measures, including bow, femoral neck, diaphyseal–condylar and version angles between reconstructed surfaces from the shape model and the intact shape reconstructed from the CT image. The reconstructions had average surface errors between 1.59 and 3.59 mm with reconstructions using the contour error metric from the AP/ML directions being the most accurate. Predictions of bow and femoral neck angles were well below the clinical threshold accuracy of 3°, diaphyseal–condylar angles were around the threshold of 3° and only version angle predictions of between 5.3° and 9.3° were above the clinical threshold, but below the range reported in clinical practice using computer navigation (i.e., 17° internal to 15° external rotation). This study shows that the reconstructions from partly available planar images using statistical shape and appearance models had an accuracy which would support their potential use in orthopaedic reconstruction.

## Introduction

Major trauma due to vehicle accidents or conflict trauma often results in severe bone damage. Orthopaedic reconstruction of these cases is especially challenging with large defects [46] or amputation. If amputation is not avoidable, the general aim is to preserve as much tissue as possible. An example of tissue salvage is in knee disarticulations for which there is a higher functional outcome than for trans-femoral amputations [32, 38]. This is particularly important for children and adolescents for whom a fusion or loss of epiphyseal plate results in a disturbance of growth causing problems in their future development and the necessity for many future interventions [20], and for patients from developing countries, where aftercare often challenges the patients and their families [3, 30].

Orthopaedic reconstruction requires knowledge of the premorbid bone shape to achieve accurate joint alignment. The shape can be used in pre-surgical planning to create cutting guides for implant placements, design patient-specific implants or be used in computer-aided systems during surgery (e.g., surgical robots, patient-specific instrumentations, augmented reality etc.). In clinical practice, the first choice to predict the intact three-dimensional (3D) geometry is to use the geometry of the contralateral side extracted from X-ray computed tomography (CT) or magnetic resonance images (MRI) [22, 40, 51]. This method is limited to cases that show no obvious asymmetries or bilateral defects and in situations, where there is ready access to costly 3D medical imaging. In developing countries and areas of armed conflict such access to MRI or CT facilities is limited.

Reconstructions of 3D bone geometry have been studied previously. The methods include reconstructions using planar X-ray images [17, 18, 28, 48], and bi-planar X-ray systems [10, 19, 41, 52] that morph a bone template to match the contour lines of the X-ray image(s). In an intraoperative navigation application, Hurvitz and Joskowicz [24] used an active appearance model to reconstruct bone surfaces from calibrated X-ray images using a C-arm system. Reconstructing 3D volumes from 2D X-ray image(s) is a challenge, and Henzler et al. [21] demonstrated the use of deep learning-based convolutional neural networks for bone reconstruction. However, the reconstruction of a bone from a partially available bone has not previously been studied, neither SSM-based nor AI-based.

For orthopaedic reconstruction, the prediction of missing parts allows for the accurate restoration of joint kinematics in the musculoskeletal system [35]. A method widely used in the literature is the prediction of missing bone parts using statistical shape models (SSM) [1, 27, 31, 34, 37, 49]. Abler et al. [1] described the reconstruction of the glenoid suited for joint reconstructions in the shoulder, Mauler et al. predicted bones of the forearm [31], Krol et al. [27] and Vanden Berghe et al. [49] reconstructed shapes of the pelvis and the hip joint.

Not only is the articular shape important, but so is the estimation of the length of long bones as this enables the restoration of normal kinematics. The measured anatomic parameters are important for pre-operative surgical planning. In forensic and anthropometric science the estimation of bone length from fragments using regression equations is frequently used. Prasad et al. [39] described regression equations on the proximal femur for a south Indian population. Solan and Kulkarni [47] related the total femur length to the length of five individual segments of the femur. Ebert et al. [16] successfully estimated the bone length from incomplete femurs using a statistical shape model presented a method for reconstruction of the bone from incomplete 3D bone, and the anatomical geometrical parameters were studied and compared to the ground truth. Salhi et al. [43] highlighted the importance to use anatomical measures to describe the accuracy of the reconstruction before clinical use. However, a wider range of anatomic measurements (e.g., femur anatomic axis, femoral neck angle etc.) were not studied in the above-cited studies.

There is currently no study that incorporates a 2D–3D reconstruction that can reliably predict missing parts of a bone shape with evaluations of the anatomical geometrical parameters that can be used for orthopaedic reconstruction and surgical planning. Therefore, in this study, the accuracy of reconstruction methods using an error metric comparing the contour of planar medical images and an error metric comparing image intensities for the shape of the femur bone from simulated planar X-ray images are evaluated for use in orthopaedic reconstruction and surgical planning.

## Results

### Surface accuracy

**Fig. 1:**
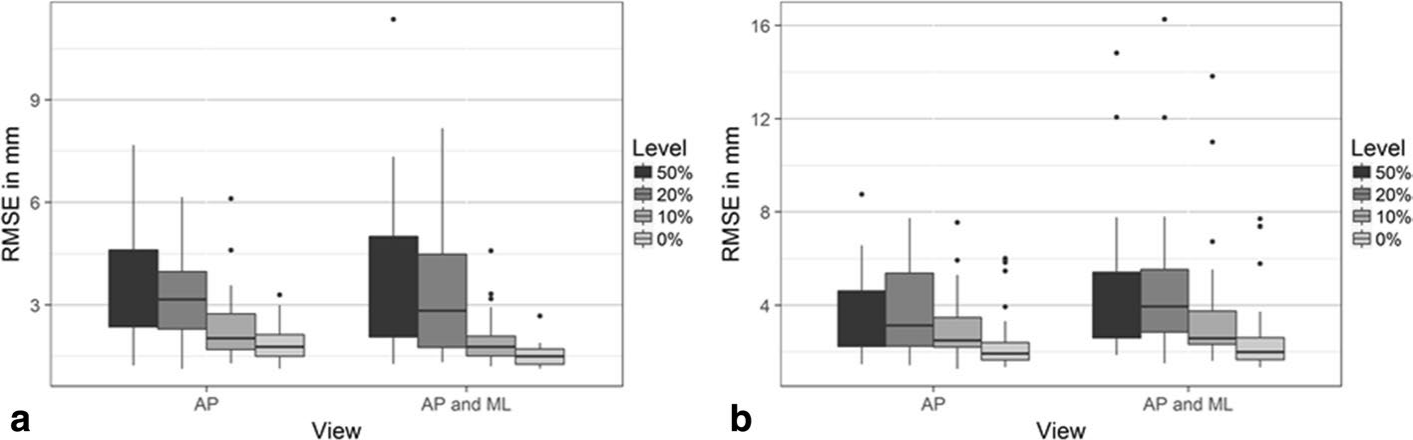
Comparison of root-mean-square errors (RMSE) between reconstructed surface and surfaces segmented from medical images using either anterior–posterior (AP) or AP and mediolateral (ML) projections, for reconstruction from bones with defect levels of 0%, 10%, 20% and 50% and the reconstruction using contour line matching (**a**) and image intensity matching (**b**)

Median root-mean-square error (RMSE) for reconstructions of geometries with defect sizes between 50% and 0% using the contour metric ranged from 3.59 to 1.77 mm for reconstructions using AP projections and from 2.59 to 1.59 mm for reconstructions using anterior–posterior (AP) and mediolateral (ML) projections. For reconstructions using the intensity error metric, median RMSE ranged from 3.03 to 1.92 mm using AP projections and 3.37 to 1.99 mm using AP and ML projections. Reconstructions using the contour error metric had significantly lower RMSE for reconstructions using AP and ML projections than reconstructions only using AP projections (*p* < 0.0005, Fig. 1a). However, for the reconstructions using the image intensity metric, the reconstructions using only projections in the AP direction had significantly lower RMSE than reconstructions using projections in AP and ML directions (*p* = 0.05, Fig. 1b).

**Fig. 2:**
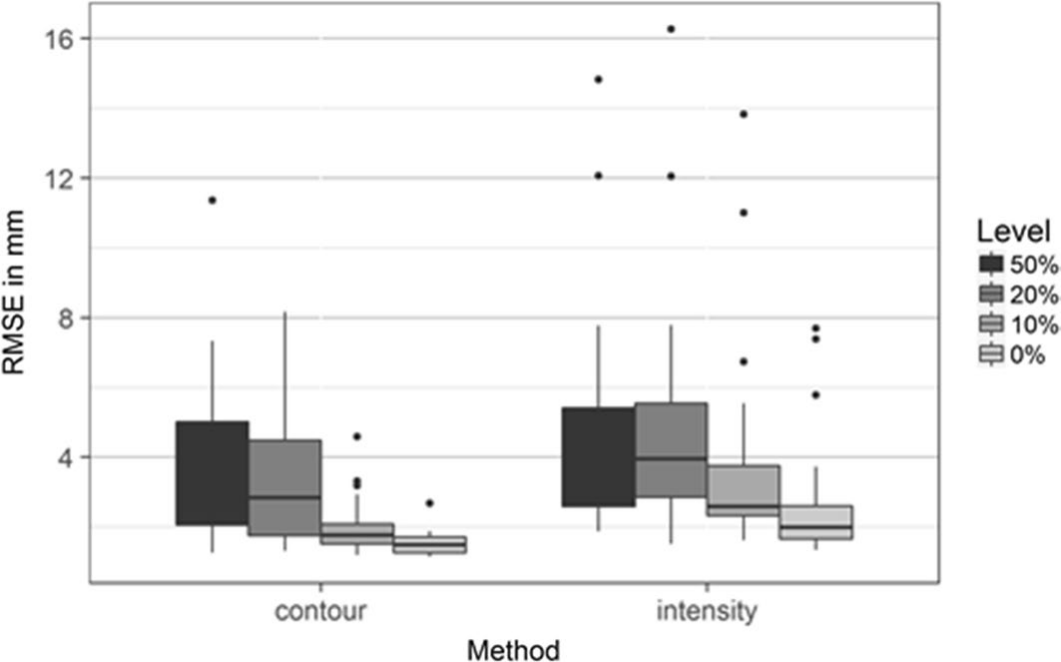
Comparison of root-mean-square errors (RMSE) between surfaces segmented from medical images and surfaces reconstructed using contour line matching and image intensity matching using projections in anterior–posterior and mediolateral directions for reconstruction from bones with defect levels of 0%, 10%, 20% and 50%

For a comparison of reconstructions using AP and ML projections over defect levels, the reconstructions calculated using the contour metric had significantly lower RMSE than reconstructions using the intensity metric (*p* < 1e−6, Fig. 2).

### Accuracy in anatomical measures

**Fig. 3:**
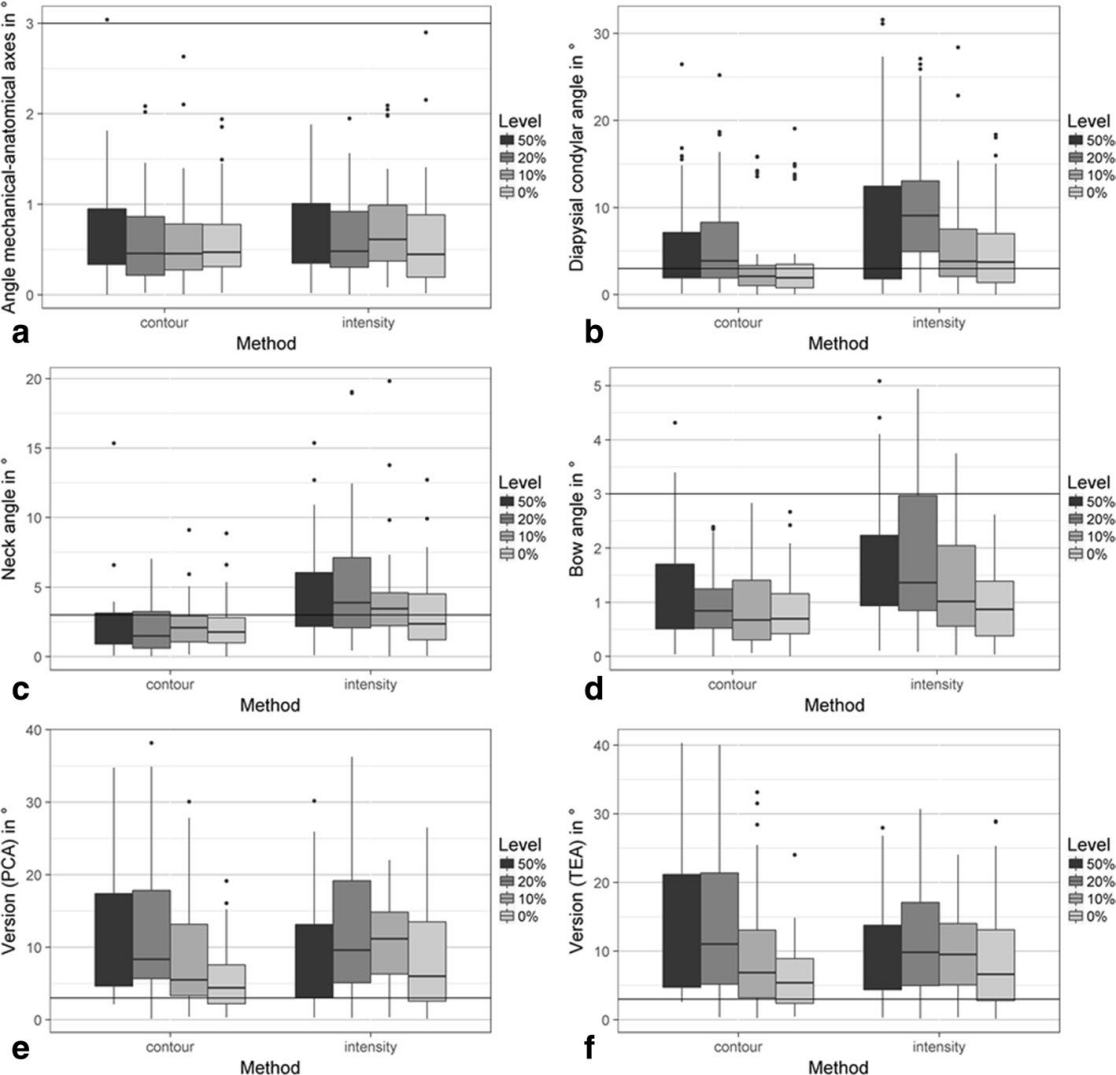
Comparison of anatomical measures between measures taken from surfaces segmented from medical images and surfaces reconstructed using the contour line and image intensity matching using projections in anterior–posterior and mediolateral direction from bones with defect levels of 0%, 10%, 20% and 50%. The measures were the angle between the anatomical and mechanical axis (**a**), diaphyseal–condylar angle (**b**), femoral neck angle (**c**), bow angle (**d**), and version angles calculated using the posterior condylar axis (PCA) (**e**) and using the trans-epicondylar axis (TEA) (**f**)

The median errors of the anatomical measures for the angle between anatomical and mechanical axes and the bow angle were small (< 2°) for reconstructions using AP and ML projections (Fig. 3a, d). For the femoral neck angle and the diaphyseal–condylar angle median errors were around 3° with errors from reconstructions using the contour metric being slightly smaller (Fig. 3b, c). The median error in version angle ranged from 5.3 to 9.3° over the various defect levels for reconstructions using the contour metric and from 6.0° to 9.1° for reconstructions using the intensity metric (Fig. 3e, f).

All average errors in anatomical measures comparing differences over different bone defect levels and error metrics are listed in Table 1. For mechanical–anatomic axis angle and bow angle, the median errors were far below 3°. The median errors for the diaphyseal–condylar angle and neck angle were around 3° and the errors increased with increasing levels of bone missing (i.e., bone defects exist from 0% and 50% of the whole bone); however, when the defects are more than 20% of the whole bone, the median error was above 3°. The mean errors for the version angles, using the posterior condylar axis (Version PCA) and trans-epicondylar axis (Version TEA) were above 3°.

**Table 1:** Comparison of differences in anatomical measures of the angle between mechanical and anatomical axis, bow angle, diaphyseal–condylar angle, femoral neck angle and version angle calculated using posterior condylar axis and trans-epicondylar axis for reconstructions using the image intensities and the contour measures with projections to the anterior–posterior (AP) and mediolateral (ML) projections

		0%		10%		20%		50%		All defect levels	
		AP	AP + ML	AP	AP + ML	AP	AP + ML	AP	AP + ML	AP	AP + ML
Intensity	Mech-anat Axis	0.58	0.45	0.65	0.61	0.88	0.48	0.70	0.73	0.65	0.58
		*p* = 0.49		*p* = 0.79		*p* = 0.2		*p* = 0.85		*p* = 0.26	
	Bow	0.67	0.87^B^	0.95	1.02	1.08	1.36^C^	1.11	1.41^B^	0.93	1.14
		*p* = 0.31		*p* = 0.47		*p* = 0.025		*p* = 0.1		*p* = 0.001	
	Diaphyseal–condylar	2.92	3.75^A^	2.75^C^	3.82^A^	4.46	9.07^A^	5.81^**C**^	7.06	3.85	5.06
		*p* = 0.77		*p* = 0.21		*p* = 0.08		*p* = 0.89		*p* = 0.14	
	Neck	2.88^D^	2.35	3.28	3.45	4.03^D^	3.88	3.62	3.80	3.47	3.40
		*p* = 0.83		*p* = 0.61		*p* = 0.95		*p* = 0.84		*p* = 0.79	
	Version (PCA)	5.80	6.00	5.86	11.15	6.06	9.58	6.25	9.49	6.06	9.16
		*p* = 0.73		*p* = 0.007		*p* = 0.09		*p* = 0.13		*p* = 0.002	
	Version (TEA)	6.28	6.61	8.05	9.50	6.97	9.81	6.35	8.28	6.68	7.94
		*p* = 0.87		*p* = 0.03		*p* = 0.11		*p* = 0.41		*p* = 0.03	
Contour	Mech-anat Axis	0.54	0.47	0.60	0.46	0.54	0.46	0.56	0.53	0.56	0.47
		*p* = 0.42		*p* = 0.34		*p* = 0.46		*p* = 0.86		*p* = 0.17	
	Bow	0.91	0.70	1.22	0.67	1.50	0.84	0.97	0.92	0.75	1.09
		*p* = 0.09		*p* = 0.06		*p* = 0.006		*p* = 0.57		*p* = 0.0006	
	Diaphyseal–condylar	1.61	1.93	2.29	2.11	2.32	3.89	2.72	3.27	2.26	2.57
		*p* = 0.77		*p* = 0.87		*p* = 0.07		*p* = 0.63		*p* = 0.17	
	Neck	2.12	1.77	1.79	2.08	2.13	1.49	2.95	1.99	2.18	1.87
		*p* = 0.25		*p* = 0.86		*p* = 0.40		*p* = 0.03		*p* = 0.05	
	Version (PCA)	6.32	4.40^A^	5.46	5.51	5.47	8.34^A^	6.71	8.38	6.28	6.57
		*p* = 0.24		*p* = 0.78		*p* = 0.06		*p* = 0.32		*p* = 0.35	
	Version (TEA)	8.00	5.38^A^	6.72	6.88	7.70	11.02^A^	8.70	9.64	7.87	7.84
		*p* = 0.10		*p* = 0.84		*p* = 0.19		*p* = 0.37		*p* = 0.85	

All values are in degrees (°), and *p* values underneath values compare values between AP and AP/ML projections. Significant differences between defect levels are indicated using superscripts

^A^*p* < 0.01

^B^*p* = 0.02

^C^*p* = 0.03

^D^*p* = 0.04

For the comparison between the number of projections (i.e., AP or AP + ML planes) used, for the bow angle, differences were significantly smaller for the contour error metric when using AP and ML projections (*p* < 0.01), but the intensity metric produced statistically significantly smaller errors when using only AP directions (*p* = 0.01). There is almost no significant difference in other anatomic parameters prediction considered in this study using single or double X-rays.

## Discussion

This study investigated if a statistical shape and appearance model (SSAM) of the femur can be used to reconstruct patient-specific bone shapes from planar X-ray images with an accuracy that enables the model to be used for orthopaedic reconstruction. Bone 3D geometry was reconstructed from simulated 2D radiographs with different levels of bone defect using two different methods (i.e., SSM and SSAM). The reconstructions were evaluated by calculating the RMSE of the reconstructed surfaces and the surfaces segmented from CT images and calculating the error of commonly used anatomical measures calculated from the surfaces.

To evaluate the methods for use in clinical applications, the precision of anatomical measures was compared to deviations reported in the literature. Knee replacement surgeries for the treatment of osteoarthritis or sports injuries typically aim for preserving the joint alignment and, therefore, the kinematics. Whereas the accuracy of the preservation is influenced by the implant type [9], the positioning of the implant has the largest effect on the kinematics. A typical threshold for studies evaluating the lower limb alignment in varus–valgus is 3° [12], Yau et al. [50] used the same threshold for the rotational alignment to calculate the success rate of joint replacement surgeries and found that over 50% of cases had angles above this threshold with values ranging from 17° internal to 15° external rotation.

In this study, the angle between anatomical and mechanical axes, and the diaphyseal–condylar angle were evaluated to estimate the accuracy of the varus–valgus alignment. Whereas median errors between anatomical and mechanical axes are well below the threshold of 3° (Fig. 3), median errors for diaphyseal–condylar angle are within the typical threshold for varus–valgus angle (i.e., 3°) when the bone defects are smaller than 20% of the whole bone. Version angles were used to estimate the accuracy of the rotational alignment. The median errors in these angles, ranging from 5.3 to 9.3°, were above the acceptable threshold used in clinical practice (i.e., 3°), but below the range reported in clinical practice using computer navigation [50].

For unicompartmental knee arthroplasties, Ng et al. [33] reported errors in implant placement between 2 and 7° in femoral rotation for a lateral implant, and Jaffry et al. [25] reported errors between 3 and 7°. These measures were evaluated using the reconstruction of the trans-epicondylar axis using pre- and post-op CT scans. The errors in diaphyseal–condylar angles and version angles reported in this study were close to these values. The benefit of the method described in this study lies in the reduced exposure to ionising radiation compared to evaluating CT images and, therefore, can be an alternative to the estimation of the 3D shapes in surgical planning for unicondylar knee replacements.

Applications using augmented reality (AR) for orthopaedic reconstructions of the glenoid reported deviations for models of the glenoid of about 2.3 mm mean error [4, 5]. Typical registration errors for head-mounted AR systems were reported between 0.8 [11] and 1.3 mm [7]. The average errors reported in this study had a comparable magnitude (Fig. 2).

The shapes reconstructed using the image intensity metric were more accurate when only using projections in the AP direction compared to using AP and ML projections (Fig. 2). Previous studies comparing reconstructions with more than one perspective for planar radiographs using an intensity error metric reported only small differences in shape accuracy [23]. In our study, the CT images were only empirically calibrated as the CT scans in the Digital Korean data set did not have phantom calibration data included, which might result in inaccuracies in the calibration and might result in variations in the image intensities. However, the exact relationship in this paper is not relevant, since the X-ray images are generated using the same relationship and, therefore, do not influence the end results. Creating a statistical shape and appearance model (SSAM) using CT image calibration using a phantom might reduce the variation which could change this behaviour. From the results of this study, reconstructions from intensity values seem less important than contour only for the prediction of bone shape. Intensity values represent the internal structure and bone density and, therefore, to a large degree the bone strength. The images for the construction of the shape model were obtained from cadavers covering a large age range and bone density. As bone density reduces with age [14, 26, 44], an SSAM for a narrow age range might produce more accurate reconstructions from matching intensity values. In addition, bone strength and shape are influenced by loading in growing bones [2]. Therefore, predictions from image intensities might be more significant in paediatric bone shape predictions if an age-specific SSAM is used.

The projection of isolated bone geometries allowed easy segmentation of the contour lines. In clinical practice, this might require more, potentially manual, work and is, therefore, a potential source of error which could affect the reconstruction accuracy. Automated methods to segment bone geometries have been described in the literature [29] which would help minimise segmentation errors. Due to the way bones overlap, such as at the hip joint and due to the patella, local differences between clinical radiographs and the simulated radiographs in this study were not taken into account. As this would only affect local regions it is assumed that it will not have large effects on the reconstruction using the matching of intensity values and only affects the reconstruction using the contour measure through the segmentation, which is not evaluated in this study. In this study, the same projection method to simulate radiographs of the target shape and the reconstructions were used. This cannot be assumed for applications in clinical practice, where images from different sources might be used. This study tried to minimise this effect using an image intensity measure which has been shown to be robust for the comparison of images from different modalities [8]. Nevertheless, the evaluation of the robustness was not part of this study and needs to be investigated separately. The RMSE were used to evaluate the surface reconstruction error in this study. This is a commonly used parameter in such studies as it assesses average errors, whereas the Hausdorff distance gives information on the maximal error. The RMSE and Hausdorff distance were closely related in our study, likely due to the rather smooth surface coming from the segmented surfaces and automated surface smoothing algorithm. Reconstruction with bone defects from 0% and 50% of the whole bone were simulated in this study; therefore, average error was prioritised to take account of the different sizes of reconstruction. Finally, the bone geometries reconstructed in this study and the shape models were accurately aligned with regard to the anatomical directions, so that the projection directions did not need to be adjusted. In clinical practice, this might not be the case and an algorithm to maximise similarities between radiograph and projection would be necessary to optimise reconstruction results. This is a research question on its own and is not addressed in this study. The X-ray image used in this study is produced from CT scans using digitally reconstructed radiographs (DRR). We used in this study linear projection with a camera distance of 1500 mm (the standard clinical distance) without applying magnification of the images, so the projected X-ray size might appear different from the true size. However, as the same DRR-based X-ray projection setup is used for the sample preparation and shape reconstruction, we believe this is unlikely to amend our study conclusions. For clinical use, the DRR parameters need to be calibrated and so further study on this point is needed.

## Conclusions

In summary, this study showed that reconstructions from 2D planar images using statistical shape and appearance models had an accuracy which would support their potential use in orthopaedic reconstruction after trauma, in examples such as a template for the reconstruction using AR systems, creating personalised instrumented guides for joint reconstruction and construction of patient-specific implants.

## Materials and methods

### Subject data

Full-body CT scans of 40 female cadaveric specimens obtained from the Digital Korean data set (http://dk.kisti.re.kr) [36] were used to create statistical shape and appearance models (SSAMs) following the method described in Nolte and Bull [34] using a leave-one-out strategy. In short, the models were created by aligning shapes using rigid body transformations calculated using an iterative closest point algorithm [6]; morphing a reference shape into all other shapes using free-form deformations [42]; creating a tetrahedral mesh of the reference shape and morphing it to all other shapes by solving a Laplace boundary condition problem as per Shontz and Vavasis [45],mapping the Hounsfield units (HU) from CT scans to the volume meshes by mapping the grey value of the closest voxel; and using a principal component analysis [13]. To allow the Hounsfield units to be read and assigned to the created shape model, we use the tetrahedral mesh to divide the shape model (i.e., surface only) into multiple “cells” (i.e., as a solid model). Since the extracted surface model shares the same coordinates with its DICOM CT images. For every single cell, with the known coordinates of the cell centroid point, we can acquire the greyscale or Hounsfield unit from the DICOM image, by repeating this for all the cells, a model can then be built with greyscale information (appearance model). The statistical shape and appearance models have previously been published [34], demonstrating compactness, generalization ability and specificity.

**Fig. 4:**
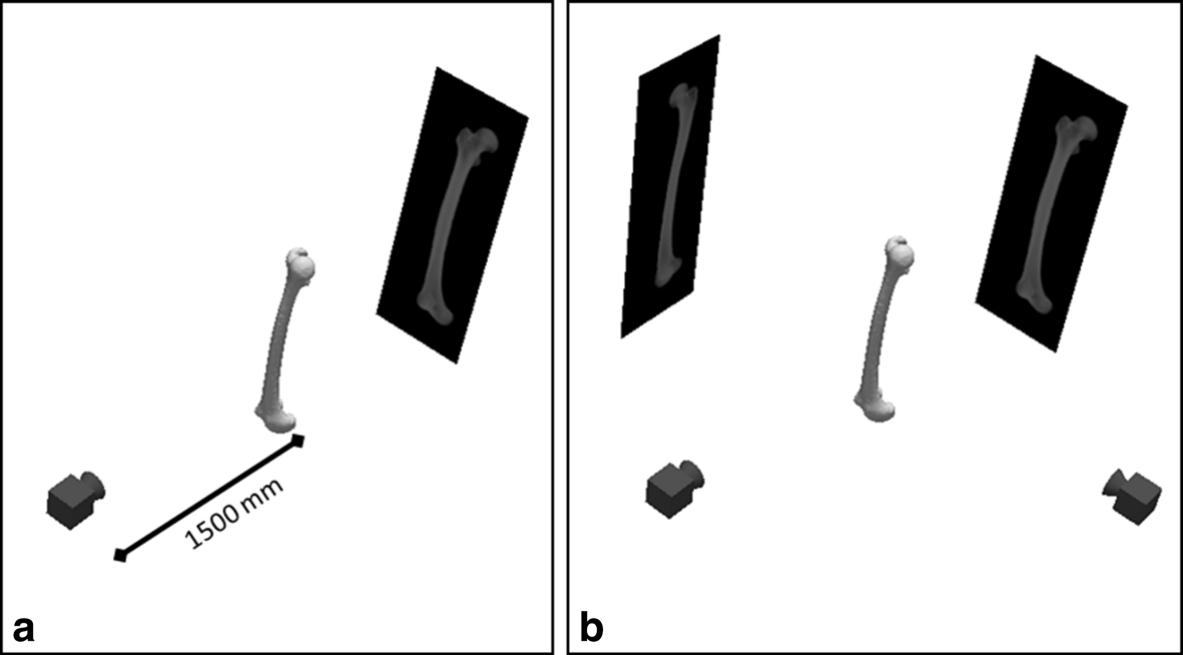
Projection directions used for reconstructions from digitally reconstructed radiographs in **a** anterior–posterior and **b** anterior–posterior and mediolateral directions

For each specimen, digitally reconstructed radiographs (DRRs) of the segmented right femur bones were created using a volume rendering method implemented in VTK (VTK 6.3.0, www.vtk.org) to simulate X-ray images. For the volume rendering, a linear transfer function describing the relationship between HU and opacity (op) values with coefficients op = 0.25 HU/1700, which was determined empirically, was used. DRRs were created for projections in the sagittal and coronal planes in anterior–posterior (AP) and mediolateral (ML) directions, respectively (Fig. 4). The projections were made with a consistent camera distance of 1500 mm to replicate a standard clinical distance.

### Shape reconstruction

The femur shapes of the 40 specimens were reconstructed from the SSAMs by calculating parameters for the modes of variation to minimise an error metric. For the reconstructions two error metrics were used: (1) quantification of the similarity of the contour of the DRR of the source and the projection of the shape model, and (2) quantification of the similarities in grey value intensities of the target DRRs and the DRRs of projections of the shape model instances. For the first metric, the contour line was extracted from the DRR of the source shape and compared to the aligned contour line of the projection of the shape model instantiation by calculating the average distance between contours. For the second metric, the grey values of the aligned images were compared by calculating a Pearson correlation coefficient of the grey values, which has been shown to be a robust measure in case the grey value ranges are not calibrated to the same scale [8]. For both measures, the images were aligned by extracting the contour lines of both images, pre-aligning to match the most proximal point of the contour lines and afterwards minimising the distance between them using an iterative closest point search algorithm [6] implemented in VTK. To determine the parameters for the modes of variation, the error measures were minimised using a Broyden–Fletcher–Goldfarb–Shanno (BFGS) optimisation algorithm for bound optimisation implemented in Python (L-BFGS-B, www.scipy.org).

### Evaluation

To simulate bone defects, the distal part of the bones on the DRRs were cropped at levels of 0%, 10%, 20% and 50% of the bone length. Shapes were reconstructed using the two methods mentioned above using DRRs in AP only or AP and ML directions. Shapes were reconstructed using 14 modes of variation. The initial estimate for the optimisation problem was determined iteratively by estimating the parameters for one mode of variation using a guess of 0.0, and iteratively using the solution as an initial guess to calculate the solution for the optimisation problem with one mode of variation more.

**Fig. 5:**
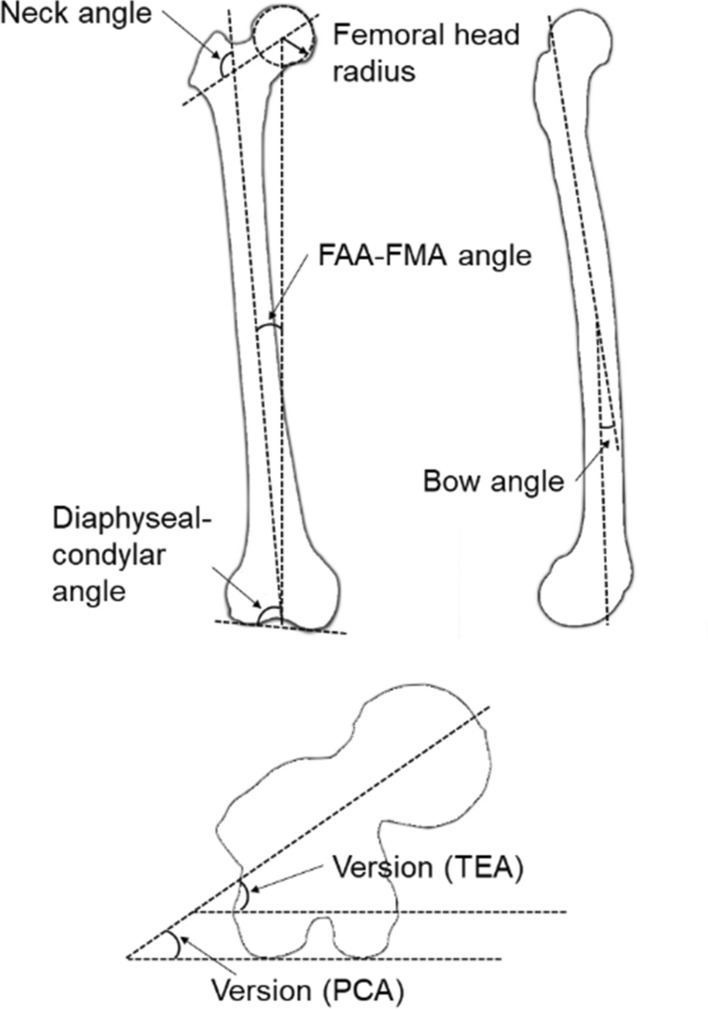
Definition of anatomical measures. Three-dimensional angles were defined as the femoral neck angle, the angle between the femoral anatomical and mechanical axis (FAA–FMA angle), the bow angle and the diaphyseal–condylar angle between the distal condylar axis and femoral anatomical axis. Version (PCA) and (TEA) were calculated from projections of the neck axis and posterior condyle and trans-epicondylar axis, respectively, onto the transverse plane

The accuracy of the reconstructed shapes was evaluated by aligning the reconstructed shapes to the segmented intact bone shapes and calculating the root-mean-square error (RMSE) between them. In addition, anatomical measures used in the literature were evaluated [15, 34]. These are used in orthopaedic interventions and are the 3D angle between two tangent lines connecting the proximal and distal end of the femoral anatomic axis (bow angle), the angle between the mechanical and anatomical axis (FAA–FMA angle), the version angle using the trans-epicondylar axis (Version TEA) and the posterior condylar axis (Version PCA), the diaphyseal–condylar angle and the femoral neck angle. Furthermore, the radius of the femoral head was estimated by fitting a sphere to points on the bone surface (Fig. 5).

### Statistical analysis

The measures for evaluating reconstructions were compared for differences between error metrics, defect level and the number of projection planes. Results were analysed using non-parametric Kruskal–Wallis tests with paired Wilcox signed rank tests in the post-hoc analysis. All tests were performed with a significance level of *α* = 0.05 using R (v3.5.1, www.r-project.org).

## Data Availability

The data that support the findings of this study are available from the corresponding author, Dr Shuqiao Xie, upon reasonable request.
